# Exploring the Role of Immersive Virtual Reality Simulation in Health Professions Education: Thematic Analysis

**DOI:** 10.2196/62803

**Published:** 2025-03-12

**Authors:** Jordan Talan, Molly Forster, Leian Joseph, Deepak Pradhan

**Affiliations:** 1 Division of Pulmonary, Critical Care, & Sleep Medicine, Department of Medicine, NYU Grossman School of Medicine, 550 First Avenue, 15th Floor, Medical ICU, New York, NY, 10016, United States, 1 2122635800

**Keywords:** virtual reality, medical education, virtual reality simulation, extended reality, simulation, VR, health professions education, health education, thematic analysis, evolving technology, qualitative study, qualitative, semistructured interviews, educational experiences, theoretical framework

## Abstract

**Background:**

Although technology is rapidly advancing in immersive virtual reality (VR) simulation, there is a paucity of literature to guide its implementation into health professions education, and there are no described best practices for the development of this evolving technology.

**Objective:**

We conducted a qualitative study using semistructured interviews with early adopters of immersive VR simulation technology to investigate use and motivations behind using this technology in educational practice, and to identify the educational needs that this technology can address.

**Methods:**

We conducted 16 interviews with VR early adopters. Data were analyzed via directed content analysis through the lens of the Unified Theory of Acceptance and Use of Technology.

**Results:**

The main themes that emerged included focus on cognitive skills, access to education, resource investment, and balancing immersion. These findings help to clarify the intended role of VR simulation in health professions education. Based on our data, we synthesized a set of research questions that may help define best practices for future VR development and implementation.

**Conclusions:**

Immersive VR simulation technology primarily serves to teach cognitive skills, expand access to educational experiences, act as a collaborative repository of widely relevant and diverse simulation scenarios, and foster learning through deep immersion. By applying the Unified Theory of Acceptance and Use of Technology theoretical framework to the context of VR simulation, we not only collected validation evidence for this established theory, but also proposed several modifications to better explain use behavior in this specific setting.

## Introduction

### Background

As technology rapidly advances in immersive virtual reality (VR) simulation, there is a growing interest among educators to develop VR simulation curricula for health professions education. However, there is a paucity of literature to guide these efforts, and there are no accepted best practices for the development or implementation of this technology. While experts anticipate the potential for VR to transform medical education [1], without a better understanding of the role VR will play in our training programs, these statements may amount to nothing more than vague future promises. Therefore, characterization of the early use of VR is imperative to clarify its evolving role and gain insights that will allow us to implement this technology to its fullest potential.

### VR Simulation Technology

Immersive VR creates a simulated environment, allowing users to “step inside” a computer-generated world and engage authentically with their surroundings [1]. VR offers several potential benefits for health professions education, including facilitating distance learning and providing training that is difficult to deliver via traditional simulation [2]. In addition, VR shows comparable educational outcomes to high-fidelity mannequin simulation with more cost-effectiveness [3-7]. Many institutions are enthusiastic about VR simulation and are already piloting or studying VR curricula [1,8] However, there is still much to learn in order to best guide the development and implementation of these curricula.

While prior research has concentrated on individual VR usage-scenarios or software evaluations [9,10], effective educational interventions require a broader understanding of the context of our learners [11]. Therefore, we must study VR user needs across a wider spectrum to guide development that aligns with the context of health professions training. By analyzing current VR educational practices, we can better identify the gaps that this technology can bridge, and move toward a consensus about how best to use VR simulation in the future. Without a better understanding of these gaps, we risk pouring resources into technology for technology’s sake—a solution looking for a problem [12].

### Study of Early Adopters

The technology adoption life cycle categorizes users into 5 groups based on their likelihood to adopt new technology: innovators, early adopters, early majority, late majority, and laggards [13]. Our study focuses on early adopters, as they represent educational stakeholders pioneering the implementation of VR within authentic educational environments and collaborating with VR innovators to adapt the technology to their needs. They therefore have expertise evaluating VR technology, but unlike the innovators, their experience is more practical than theoretical.

### Unified Theory of Acceptance and Use of Technology

**Figure 1.**
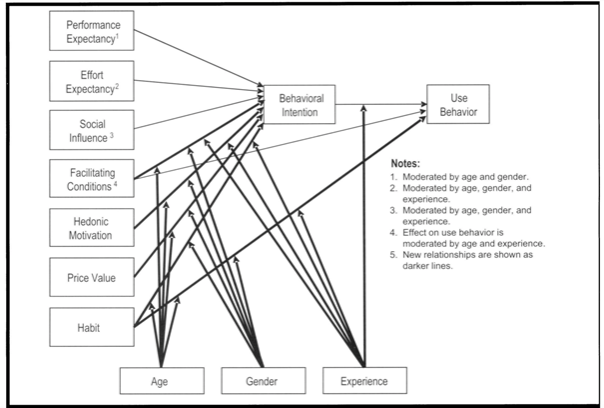
The UTAUT2 from Venkatesh et al [16], used with permission. UTAUT2: Unified Theory of Acceptance and Use of Technology 2.

The Unified Theory of Acceptance and Use of Technology (UTAUT) explains factors that affect the adoption of new technologies and predicts future technology use [14]. UTAUT provides a robust theoretical framework for understanding the drivers incentivizing early adopters to embrace VR as an educational strategy. The original theory described 4 constructs as direct determinants of technology usage behavior (Figure 1): performance expectancy (user expectation that the technology improves performance), effort expectancy (ease associated with using the technology), social influence (user perception that others believe they should be using the technology), and facilitating conditions (organizational and technological infrastructure for technology implementation). These determinants are modified to varying degrees by user gender, age, or experience [14]. Extensively applied across multiple fields for assessing new technologies [15], the UTAUT was expanded to the Unified Theory of Acceptance and Use of Technology 2 (UTAUT2) with 3 additional constructs: hedonic motivation (pleasure derived from using the technology), price value (perceived cost of the new technology), and habit (degree of automatic use of the technology) [16].

With validity evidence across multiple fields since 2003, UTAUT has become one of the most developed and intensive models to test new technologies [15]. In addition to being well-validated in multiple settings, UTAUT is an ideal theoretical framework to explore and better understand thoughts and behaviors associated with the use of VR simulation technology. While other theoretical frameworks have been used to approach VR simulation research, few will allow the isolation of factors associated with VR specifically rather than those that apply to simulation in general. Even fewer might facilitate the prediction of its use in the future. For example, constructivist learning theory has been applied to VR simulation because learners can manipulate a problem and construct learning from active participation in an engaging experience [17]. Experiential learning has also been used to contextualize VR simulation because it provides a safe and forgiving training environment that facilitates learning by doing [3]. However, these theoretical frameworks serve better to characterize simulation in general rather than to focus on the specific experience provided by VR technology. The use of UTAUT2 on the other hand provides a structured framework by which we can distinguish the features of VR technology from other modes of simulation, and by which we can attempt to predict its future use.

Prior VR research using UTAUT2 focuses primarily on understanding learner experience and learner acceptance [18,19]. While these concepts are critical for the successful adoption of evolving technological innovations [20], we must progress further by investigating how VR simulation can address specific educational needs and gathering validation evidence for its most effective future role in the evolving landscape of health professions education.

### Study Aims

To fill this gap in our understanding, this study interviews early adopters of VR simulation in health professions education, with the following aims: (1) characterize how early adopters are adapting VR to meet their educational needs, (2) define the educational problems or gaps that early adopters are trying to address with VR, and (3) explore factors influencing the ability of early adopters to meet their needs with VR.

## Methods

### Ethical Considerations

This study was approved by the New York University Langone Institutional Review Board (22‐01346). Informed consent was obtained from all study participants, and participants had the ability to opt out or withdraw from the study at any time. Interview transcripts were deidentified for confidentiality. Participants were not compensated.

### Study Design

This is a qualitative study using thematic analysis of semistructured interviews. The research methodology is directed content analysis, starting with a limited code book of 7 a priori codes defined through the lens of the UTAUT2 theoretical framework, followed by an exploratory coding phase [21,22]. The research paradigm is postpositivist. Reporting was completed following the Standards for Reporting Qualitative Research guidelines [23].

### Semistructured Interview Guide

We iteratively developed a semistructured interview guide based on our research questions and grounded in the UTAUT2 theoretical framework [24,25] (Multimedia Appendix 1). The interview guide was piloted with local stakeholders to ensure capture of meaningful data within the 45-minute interview timeframe.

### Recruitment and Sampling

We recruited educational stakeholders who were identified as “early adopters” of immersive VR simulation technology. Inclusion criteria were experience educating, implementing, or researching with VR. Exclusion criteria included technology developers without educational practice experience, and participants with experience limited to 360° video, augmented reality, or nonsimulation immersive learning.

The first 3 participants were recruited as a convenience sample, as they were known to our research team based on their work with the American College of Chest Physicians to develop and pilot an immersive VR simulation program teaching endotracheal intubation. These participants were recruited as an entry into the community of VR early adopters, with subsequent recruitment by snowball sampling. We sought to map the terrain of VR use-cases by recruiting for maximal diversity. We asked if participants could identify additional early adopters who had different experiences (ie, worked with a different company, in a different learner setting, at a different institution, or who had differing perspectives on VR technology). We estimated a sample size of 12‐18 interviews. Data were iteratively analyzed for thematic saturation, and recruitment was terminated upon achieving saturation of meaning [26].

### Interviews and Data Analysis

Each participant completed a 45-minute semistructured interview via Zoom videoconferencing. Interviews were audio-recorded and transcribed verbatim into a written document via Speechmatics software with manual verification. Transcripts were imported to ATLAS.ti (ATLAS.ti Scientific Software Development GmbH) web, which was used for iterative qualitative data coding and analysis. First-round coding was performed via an a priori coding template corresponding to the UTAUT2 domains. Any additional codes used process coding and descriptive coding. All codes were approved by 2 independent reviewers (JT and DP) with deliberation over any discrepancies. Second-round coding then checked all codes against the initial coding template, collapsing as necessary to capture any new domains not described by the UTAUT2 framework. Field notes and memos were maintained by both reviewers. Themes were identified and their interrelationships characterized [27]. Themes were then shared with study participants via member checking to ensure the accuracy of our analyses.

### Reflexivity

JT and DP are Pulmonary/Critical Care Medicine physicians. JT has worked with technology companies and educational technologists researching immersive VR simulation, but is relatively suspicious of new technology unless it fulfills a specific need. DP is also an early adopter, who is a self-described “gamer” and owns a VR headset for recreational use. JT, DP, and MF are simulation educators at New York University. All authors kept memos to practice reflexivity throughout this study’s period.

## Results

### Overview

We completed 16 semistructured interviews. Coding saturation occurred after 11 interviews and thematic saturation after 12 interviews. Four additional interviews were completed to ensure saturation of meaning [26]. Participant demographics are described in Table 1. Our study population included early adopters from diverse health professions whose educational interventions targeted the following groups of learners: physician trainees (premedical students, medical students, residents, and fellows), advanced practice providers (nurse practitioners and physician assistants), nurses and nursing students, respiratory therapists, pharmacists, and emergency service members (emergency medical technician students and paramedical students).

**Table 1.** Demographics of interview participants (N=16).

Demographics of interview participants	Values (n)
Gender	
Male	10
Female	6
Age (years)	
31‐50	8
51‐65	3
>65	3
Unknown	2
Technology adoption life cycle group	
Innovator	6
Early adopter	6
Early majority	3
Unknown	1
Geography	
Northeast (United States)	3
Midwest (United States)	5
South (United States)	4
West (United States)	3
Canada	1
Setting	
Urban	12
Suburban	4
Rural	0
Health profession	
Advanced practice provider (nurse practitioner or physician assistant)	1
Emergency medical service (paramedics or emergency medical technicians)	1
Health care education technologist	1
Nurse	3
Physician	8
Anesthesiology	2
Cardiology (pediatrics)	1
Emergency medicine (adult)	2
Armed forces	1
Emergency medicine (pediatrics)	1
Internal medicine	1
Pulmonary and critical care medicine	2
Respiratory therapist	1

### Coding and Themes

**Figure 2.**
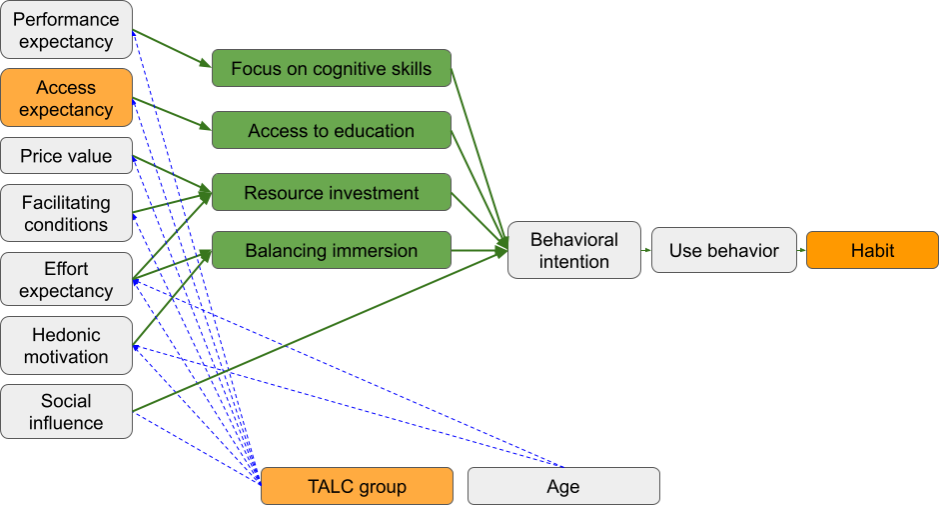
Thematic map of results. The thematic map illustrates our results and changes we have made to the theoretical framework. Themes (green boxes) are superimposed between each UTAUT construct and the resulting behavioral intention. The new addition of access expectancy is highlighted in orange. Green arrows illustrate which theme most strongly relates to which UTAUT construct. Blue arrows represent effect modifying relationships. TALC: technology adoption lifecycle; UTAUT: Unified Theory of Acceptance and Use of Technology.

First-round coding generated 38 unique codes: 7 from the a priori coding template corresponding to UTAUT domains, and 31 new codes via process and descriptive coding. Four themes were identified and examined for their interrelationships. The resulting synthesis and validation evidence for the UTAUT2 framework are depicted in a thematic map (Figure 2).

### Theme 1: Focus on Cognitive Skills

Study participants focused on VR simulation for the development of cognitive skills, including communication, teamwork, clinical reasoning, situational awareness, and interdisciplinary skills, occasionally referred to as “soft skills.”

*It’s really just about talking to each other, right? And sharing that mental model…I think that’s where you can really benefit from VR because it’s not really about the tasks you're doing. It’s how are you communicating…I think if you look at a lot of the sentinel events or the near misses that happen, it’s based on communication.*

[Participant #4]

For procedural skills, the role of VR simulation was limited to building procedural knowledge or situational awareness.

*It does help you remember the different steps. You know, don't forget the suction at the head of the bed or, you know, [we will] have the patient vomit…and maybe they won't forget it now.*

[Participant #4]

However, participants found the teaching of fine psychomotor skills, such as laryngoscopy or peripheral intravenous placement, to be limited in VR.

*We knew that you cannot teach the fine motor skills of intubating a patient in virtual reality. It is very difficult to do with the kind of tools that are available right now. And so it was more about the thinking…I truly believe that the mental process of approaching an airway is just as important, if not more important, than the fine tuning of technical skills.*

[Participant #1]

The most common barrier to teaching psychomotor skills in the VR environment was haptic technology, noted unanimously by every interview participant.

*What it doesn't do well? One is teaching people how to do things that require them to use their hands and fine motor skills, even just like how to use tools. It’s really challenging to teach somebody how to hold a laryngoscope, how to hold the endotracheal tube…I think the big limiting factor is the fact that you have to use controllers because the controllers only work a certain way. You must hold it this way. These are the few buttons that you have. You're not using your hands the way you would in real life.*

[Participant #1]

In addition to limitations in simulating authentic tools, participants noted limitations in simulating the weight or feel of human anatomy necessary to learn fine psychomotor skills.

*You put the [laryngoscope] blade in their mouth, the vocal cords show up on the screen and you just drop the tube and it just clicks right in. But right now, we don't have…that feedback where you feel your scope in your hand or you feel the weight of the jaw when you're going to lift up.*

[Participant #8]

Many participants discussed the development of haptic gloves which can allow users to simulate touch and contact experiences. However, most found current solutions either cost-prohibitive or inadequate.

*I don't see that type of fine motor feedback, you know, where I know how to put the needle into an arm for an IV, for example. That’s not going to happen for - I would say that’s decades away. Easily. I don't think there are good solutions right now in even the most expensive labs with experimental haptics for that.*

[Participant #16]

Therefore, to teach psychomotor and procedural skills, participants turned to other forms of simulation technology such as mixed reality or mannequin simulation. Several participants offered learners a blended experience, using VR to create an immersive scenario, followed by a task trainer to simulate any necessary fine motor tasks.

### Theme 2: Access to Education

The ability of VR simulation to facilitate distance learning was seen as a significant driver of use behavior for most participants.

*We wanted to break down the barriers of requiring learners to physically come to a place to get this type of education. We want this education to be deliverable over long distances to people in other parts of the world.*

[Participant #1]

In areas without the resources for a high-fidelity mannequin-based simulation laboratory, VR was seen as expanding access to high-quality simulation learning from expert educators.

*Places that don't have simulation labs and all of those resources…available at academic medical centers, at [professional society] headquarters…But outside of large centers or hospitals that have access to a sim lab - and I think probably the majority of hospitals in the country do not - those hospitals don't have access to that type of education.*

[Participant #1]

VR simulation was also used as a solution to reduce the cost, inconvenience, and sometimes danger associated with traveling to simulation centers.

*The ability to do remote simulation at a much lower cost than requiring travel, that’s a huge benefit…If you've got employees spread across the country, even across the state - and I'll use Wyoming, for example… everything’s 8 hours away. It’s icy half the year. So if…you've got students all over the state that are part of your paramedic program, and you have these guys driving throughout the winter to come to your simulation center. Like, what are the chances of something bad happening there? Pretty high to be honest.*

[Participant #8]

Even in centers with existing high-fidelity simulation laboratories, participants found a role for VR in facilitating collaboration and standard-setting at a national or international level.

*As people adopt these headsets…somebody from [University] could do the same ACLS training as somebody at [University] and it would be the same across institutions. And there’s crosstalk - so different learning points and different perspectives and shared information and shared values in terms of education.*

[Participant #6]

Finally, VR simulation also expanded access to education during the COVID-19 pandemic, responding to the need for social distancing, and significantly accelerating VR adoption.

*If heaven forbid there was another pandemic, now we are set up that. If our students were at home on lockdown, as long as they had a headset, their learning would not be interrupted.*

[Participant #13]

Overall, VR afforded a distance learning advantage, providing equitable access to high-quality simulation education to centers in diverse settings and learners in adverse scenarios.

### Theme 3: Resource Investment

Implementation of a VR simulation curriculum required extensive resources, particularly upfront costs (funding and time commitments).

*There is a capital purchase that has to be made just for the equipment itself. But…how do you develop that program in a manner that somebody is not spending tons and tons of time to bring one little educational module to fruition.*

[Participant #3]

These upfront costs also related to the process of cocreation with technology companies.

*There was generally some frustration during the build process because we're all clinicians and we're like, ‘yeah, this thing needs to be this way’. And you're trying to communicate that with someone who has no medical experience and is a software programmer… We speak one language and they speak a different language, and there was some inability to communicate that effectively.*

[Participant #3]

Once the programming was complete, participants also described an ongoing cost to maintain the software through updates or licensing.

*Keeping these things alive is really…the cost to maintain software…for servers and engineers and updates and things like that. So without some sort of continued funding from somewhere, it will become a useless pile of code as soon as the next [operating system] update hits.*

[Participant #9]

The investment required to develop novel VR programming was frequently more resource-intensive than anticipated, and the risk of failed investment was wasted time and money.

*I’ve seen many cases of projects that are developed and they're just abandoned…I would walk into my office every morning and I had a stack of 16 boxes of headsets we didn't use.*

[Participant #10]

Therefore, participants wanted more opportunities for creative collaboration and sharing of software programs. However, some felt limited by the current state of technology.

*There’s no great way to share content yet. So a lot of stuff is just getting reinvented over and over again, which is a really expensive way to do things.*

[Participant #12]

Others felt restricted by the current incentives within the VR marketplace, concerned by compatibility between different software or hardware companies.

*There needs to be an ability for me to use multiple vendors within my one headset without having to pay millions of dollars to do so…I don't know about everybody’s budget. On my budget, I cannot afford to pay four different guys for completely different programs.*

[Participant #8]

Generally, participants desired to use pre-existing software that was universally relevant for multiple institutions and multiple users, and compatible with a variety of hardware.

### Theme 4: Balancing Immersion

The immersiveness of VR was a powerful experience associated with learner enjoyment.

*Being in virtual reality is an immersive experience, and it’s just hard to describe in words until you try it. But when people try it, it’s like seeing a new color.*

[Participant #1]

At its best, immersion increased learner presence, stimulated intellectual curiosity, and accelerated learning.

*When you go in and you see an environment in 3D that looks exactly like your cardiac ICU…you immediately have a ‘wow’ thing. And what I love about that is immediately when I start this scenario, I never really hear like, ‘Wait, what do you want me to do? Are we starting now? Is the patient supposed to have pulses?’…It’s so immersive that people immediately feel like they're in a football game and it’s kickoff.*

[Participant #7]

Participants also valued VR immersion for minimizing distractions more than other simulation technologies.

*The thing that’s nice about virtual reality is you put the headset on and that’s what you're doing, right? So you're not looking at your phone or checking your email while someone’s trying to teach you.*

[Participant #3]

However, immersion could also create extraneous cognitive load, detracting from learning. Participants described unnecessary environmental elements that distracted from learning objectives, along with some tasks that were frustrating to simulate in VR.

*The picking up of items in the ICU was difficult always…With the limited controller toggles, it was not always intuitive how to pick something up. And even when they told you what to do, it still sometimes fell on the floor and stuff like that.*

[Participant #2]

Sometimes immersive scenarios became more about navigating the VR environment than mastering intended learning objectives.

*You're never going to drop some instrument on the tray 6 times…Like is the goal to learn to pick the scope up, or to [learn the procedure]?…I think a lot of people try and make the virtual world exactly like the real world…but I think you have to simplify the haptics…If it’s just so frustrating because you're an intensivist and you can't pick up the needle drivers, then forget it. There shouldn't be a five minute learning curve on how to pick up needle drivers, right?*

[Participant #12]

Participants found an ideal immersive balance when the virtual world accomplished the intended learning objectives, but was not overly complex to create frustration in navigating the environment. In this way, there was constructive alignment between the intended learning outcomes and the virtual learning activities.

### Validation Evidence for the UTAUT2 Framework

Codes were confirmed for each previously described construct within the UTAUT2 framework [16]. Performance expectancy was the most frequently coded driver for use intention with VR technology. We also found age to modify the effect of certain constructs, with the younger generation more easily adapting to VR technology (effort expectancy) and demonstrating greater VR learning enjoyment (hedonic motivation).

*I think the current generation of learners is…becoming more and more comfortable with virtual reality. So I think the buy-in of our new generation of learners is going to be really quick…And so I think they're going to help drive the need for this type of education.*

[Participant #5]

The UTAUT2 theoretical framework was able to explain patterns in use behavior and intention related to VR simulation, and provided conclusions relevant to educational practice. However, we modified the UTAUT2 model, most notably adding “access expectancy” as an independent driver of behavioral intention. The importance of distance learning, expanded access, and equity in educational experience was sufficient to qualify as an independent construct. To illustrate its relative importance, there were more instances of coding for access expectancy than hedonic motivation, habit, or social influence. This may reflect that the UTAUT was initially described in the individual consumer marketplace while “access expectancy” applies more to the context of the educational technology marketplace. Further research would be necessary to explore this hypothesis.

The other notable change was seen in the UTAUT modifier “gender.” There were no instances of coding applicable to gender by either independent reviewer, and themes identified did not differ by participant gender. We found no signal for gender as a modifier of any UTAUT2 construct. We suggest that this is a reflection of both time and context. The initial publication of the UTAUT was in 2003, wherein it was discussed that effort expectancies may be more salient to women than men, and that women may be more sensitive to others’ opinions than men [16]. We believe this contextualization of gender roles and social norms to be antiquated and due for revision. Furthermore, in this cohort of career medical educators, we found no differences in motivating factors between men and women related to their intention or use behavior with simulation technology. Therefore, we eliminated “gender” from our thematic map.

## Discussion

### Principal Findings

Participating early adopters have adapted their use of VR to meet specific educational needs. Whether it be the need for distance learning during the pandemic, the need to bridge geographical or institutional divides, or the need for wide dissemination of teaching to address gaps in knowledge or skills, early adopters are implementing VR as a method to expand access to high-yield educational interventions. In terms of the role that VR served among the studied population, it was used primarily to teach cognitive skills as opposed to psychomotor or procedural skills. The most common factors that affected how successful any given implementation of VR would be is related to how educators managed their resources (funding, time, and design effort) and the degree to which they were able to foster learning through deep immersion.

The results of this study establish drivers of use behavior, providing practical insights into the educational gaps that VR might address in the future. Furthermore, this study contributes validity evidence for the UTAUT framework in studying the evolving role of immersive VR simulation in health professions education. This study represents an advancement to the literature in this field as it encompasses a wider variety of VR use cases than prior work, and it uses a well-validated theoretical framework to reflect on the perspectives of a diverse population within the health professions education community.

### Implications for Future Research

Based on our findings, we synthesize a set of research questions that may help define best practices for future VR development and implementation (Table 2). We also list example study ideas corresponding to each research question in order to provide additional context and encourage reflection. These examples are not meant to be comprehensive or prescriptive, but rather to demonstrate how researchers might approach these questions with a variety of different methodologies and paradigms that could advance the literature in this field.

**Table 2.** Suggested research questions for immersive VR^a^ simulation technology.

Themes from this study and suggested research questions		Example future research study
Focus on cognitive skills		
	How can we best implement immersive VR simulation given its strength in teaching cognitive skills (eg, communication, teamwork, clinical reasoning, situational awareness, and interdisciplinary skills)?	Multi-institutional study comparing 2 different VR implementation methods and using a validated assessment for cognitive skills
	What innovations can improve the teaching of fine psychomotor tasks in the VR environment?	Validation study using novel haptic gloves for VR simulation and assessing learning outcomes
Access to education		
	How can VR be most effectively leveraged to provide distance learning?	Mixed methods (quantitative or qualitative) needs assessment for distance simulation learning in post-COVID health professions education
	How can VR be used as a tool to create equity of educational experience?	Comparative study of learner outcomes at highly resourced centers versus resource-limited training programs for VR simulation
	How can VR facilitate collaboration on a larger scale (eg, national or international)?	Descriptive study demonstrating feasibility of an international VR curriculum offered by a professional society
Resource investment		
	What are the upfront investments and preparation processes necessary to start a new VR simulation program?	Focus group study of early adopters with concentration on preparation and upfront costs for establishing a VR simulation program
	How can we increase availability and decrease barriers for using pre-existing VR software programs?	Thematic analysis of focus groups after piloting a VR multi-case library targeting undergraduate medical education learners
	What processes facilitate the creation of novel VR software that is relevant to external users, institutions, and learner groups?	Systematic review and subsequent guideline development project to describe best practices in creation of VR curricula
Balancing immersion		
	How can we achieve sufficient immersion to accomplish intended learning objectives without creating extraneous cognitive load and frustrating part-tasks?	Comparative study of learning outcomes in a high-fidelity versus low-fidelity VR environment

a VR: virtual reality.

Much ongoing VR simulation research focuses on demonstrating that VR is equally or more effective than traditional simulation modalities [3-6,28]. While this is an important question, it risks overshadowing other questions that are raised by early adopters in this study: how might we improve the ability of VR technology to teach psychomotor skills? How can we use VR simulation to create equity between learner populations? What solutions exist for shared and collaborative creation of VR software? How can we leverage the incentives of the marketplace for VR technology companies? These questions could significantly impact the future use of this technology in health professions education.

### Study Limitations

This study has several limitations. First, early adopters tend to be optimistic about the advantages of new technologies, sometimes underemphasizing associated challenges. To account for this bias, we designed our interview guide with prompts targeted equally toward the advantages and challenges of VR technology, and we practiced reflexivity among this study’s team to appreciate the effects of any personal biases. Future studies should examine perspectives from early majority, late majority, and laggards, but these groups are not yet readily identifiable.

Second, this study used nonprobability sampling, which harbors potential for bias toward participants with similar experiences and perspectives. However, the small size of the VR educator community limits the feasibility of random sampling. We therefore attempted to compensate by seeking participants with diverse experiences, working with different software applications, different VR companies, or different learner populations. To further assure accuracy and freedom from bias, future studies should attempt triangulation of this data, for example via data source triangulation using focus groups or via theory triangulation, analyzing this data through a different theoretical lens [29].

Third, while we targeted diversity, all health professions were not represented. Our sample included only individuals from the United States and Canada, and participants from rural workplace settings were underrepresented. These considerations may be important, particularly if geography is found to independently affect use behavior.

Finally, regarding the UTAUT modifier “experience,” our sample size was inadequate to analyze its role as a modifier of use behavior. Therefore, we did not include experience in our thematic map and further research will be necessary to explore how experience may affect use behavior with VR simulation.

### Conclusion

We used the UTAUT2 framework in a directed content analysis using semistructured interviews to investigate the role of immersive VR simulation in health professions education. We identified 4 key themes elucidating use behavior related to VR simulation, suggesting its optimal applications include teaching cognitive skills, expanding access to educational experiences, offering a collaborative repository of relevant simulation scenarios, and enhancing immersion for intended learning objectives. These themes may help to inform best practices for the future development and implementation of immersive VR simulation programs.

As immersive VR simulation technology continues to evolve in health professions education, the VR educator community will continue to grow alongside the rapid technological advancements. Therefore, defining best practices for integrating this technology into training programs is critical. Future research should focus on leveraging VR simulation’s unique capabilities as compared to traditional simulation modalities.

## Supplementary material

10.2196/62803 Multimedia Appendix 1 Semistructured interview guide.

## Abbreviations:

UTAUT: Unified Theory of Acceptance and Use of Technology
UTAUT2: Unified Theory of Acceptance and Use of Technology 2
VR: virtual reality
