# Supplementary material for: Exploring the Role of Immersive Virtual Reality Simulation in Health Professions Education: Thematic Analysis
Source: JMIR Med Educ. 2025 Mar 12;11:e62803. doi: 10.2196/62803 (PMC11922489; doi:10.2196/62803)
Supplement: Multimedia Appendix 1 [file mededu-v11-e62803-s001.docx]

Thank you for taking the time to speak with us about immersive virtual reality simulation technology. We are interested in learning more about what factors are driving the development of this technology and the settings in which it is being used. This interview is for research purposes only. The interview will be audio recorded and transcribed to writing, but your identity will not be stored in connection to the interview and your identity will not be included in any publication associated with this research. We will also de-identify any other named person, company, or institution. This interview is voluntary and you may stop at any time or decide not to answer any of these questions. Is it okay if we get started?

===================================================================

Great, these first few questions will be about your **attitudes towards technology** in general and your **attitudes towards VR technology**.

1. Tell me about yourself and how you got interested in VR?

2. Can you tell me a little bit about your attitude towards new technologies in general?

3. Some people might be more accepting than others of new technologies. Other people might be hesitant to adopt new technologies for a variety of reasons. Where do you see yourself on this spectrum?

========================================================================

These next few questions will be about your **experience with VR technology** and the **project that you are developing/piloting/researching** with [company].

1. Can you tell me a little about your experience with VR technology?
2. Tell me about your current project developing/piloting/researching VR with [company].

Follow-up questions about the project:

- 1. What kinds of learners are you targeting with your project?
  2. How does your project go about debriefing after the simulation? Why do it this way?
  3. Why do you think VR would work particularly well in this setting?
  4. Why did you choose VR? *Explore UTAUT Categories in follow-up questions*

| ✓ | **UTAUT Category** | **Follow-up Questions** |
| --- | --- | --- |
|  | Performance Expectancy | Do you think VR sim works for teaching? Why or why not?  What is it specifically about VR that makes it work better/worse than traditional simulation? |
|  | Effort Expectancy | Why is it hard or easy to use?  How do you think the difficulty/ease of use will affect its future adoption? |
|  | Social Influence | How have you been influenced by peers in your use of VR?  How has this affected your use of the technology?  How do your peers react to your use of VR? How has this affected your use? |
|  | Hedonic Motivation | Is VR cool? Enjoyable?  How does that affect its adoption? |
|  | Facilitating Conditions | What resources are needed to successfully adopt this technology?  How will that affect its widespread adoption and why? |
|  | Price value | What is the price value of VR compared to traditional simulation?  Are you willing to pay for headsets and software in your institution? Licenses that must be renewed? Why or why not? |
|  | Habit | Could VR sim become a standard part of medical education for health professions trainees in the future?  Why or why not? |

- 1. Have you thought about using any alternative technology to VR? Why or why not?
  2. If you could see a successful future for the implementation of this project, what would that look like?
  3. Is there anything you’re worried about in implementing your program?

1. What is it in particular that VR brings to your project that you can’t achieve with other simulation technologies such as mannequin simulation?

========================================================================

These final few questions will be about how you see the **future of VR technology**. There is no right answer to any of these questions, but we ask you to give your personal perspectives.

1. What do you think VR specifically as a technology brings to simulation as an advantage?
2. What is it that VR doesn't do well?
3. What are some of the challenges or barriers to implementing VR technology?
4. What might drive a certain learner population to want or need to use VR simulation?
5. In what settings do you think VR might fill a specific need? In other words, where would VR provide an advantage that we would be unable to achieve with other technologies?

1. Could VR sim become a standard part of medical education for [trainees] in the future? Why or why not?

========================================================================

Thank you for participating in this interview. We appreciate your time. We want to invite you at this time to add anything else that you would like to say.

Did you get across everything that you wanted to say about yourself and about VR?

Okay, if there is nothing else at this time, we will end the interview. Thank you again.
